# Long-Term Grow-Out Affects *Campylobacter jejuni* Colonization Fitness in Coincidence With Altered Microbiota and Lipid Composition in the Cecum of Laying Hens

**DOI:** 10.3389/fvets.2021.675570

**Published:** 2021-06-18

**Authors:** Hiroshi Asakura, Tatsuya Nakayama, Shiori Yamamoto, Kazuki Izawa, Jun Kawase, Yasushi Torii, Satoshi Murakami

**Affiliations:** ^1^Division of Biomedical Food Research, National Institute of Health Sciences, Kawasaki, Japan; ^2^Department of Computer Science, Tokyo Institute of Technology, Meguro City, Japan; ^3^Department of Bacteriology, Shimane Prefectural Institute of Public Health and Environmental Science, Matsue City, Japan; ^4^Department of Animal Hygiene, Tokyo University of Agriculture, Atsugi City, Japan

**Keywords:** *Campylobacter jejuni*, laying hen, long-term breeding, gut microbiota, lipidome

## Abstract

*Campylobacter jejuni* is one of the leading causes of gastrointestinal illness worldwide and is mainly transmitted from chicken through the food chain. Previous studies have provided increasing evidence that this pathogen can colonize and replicate in broiler chicken during its breeding; however, its temporal kinetics in laying hen are poorly understood. Considering the possible interaction between *C. jejuni* and gut microbiota, the current study was conducted to address the temporal dynamics of *C. jejuni* in the cecum of laying hen over 40 weeks, with possible alteration of the gut microbiota and fatty acid (FA) components. Following oral infection with *C. jejuni* 81-176, inocula were stably recovered from ceca for up to 8 weeks post-infection (*p.i*.). From 16 weeks *p.i*., most birds became negative for *C. jejuni* and remained negative up to 40 weeks *p.i*. 16S rRNA gene sequencing analyses revealed that most of the altered relative rRNA gene abundances occurred in the order *Clostridiales*, in which increased relative rRNA gene abundances were observed at >16 weeks *p.i*. in the families *Clostridiaceae, Ruminococcaceae, Lachnospiraceae*, and *Peptococcaceae*. Lipidome analyses revealed increased levels of sterols associated with bile acid metabolisms in the cecum at 16 and/or 24 weeks *p.i*. compared with those detected at 8 weeks *p.i*., suggesting that altered microbiota and bile acid metabolism might underlie the decreased colonization fitness of *C. jejuni* in the gut of laying hens.

## Introduction

*Campylobacter jejuni* is one of the most reported foodborne pathogens to cause gastrointestinal illness worldwide (1, 2). Similar to the western countries, foodborne campylobacteriosis accounted for 27.0% of the total cases of foodborne gastrointestinal illness reported in 2019 in Japan (3). Several source attribution studies have provided increasing evidence that poultry and poultry products are among the main sources of human campylobacteriosis (4–6), which highlights the necessity to control this pathogen in poultry and poultry products.

The prevention of *C. jejuni* invasion and spread in poultry farms is recognized as one of the key issues for reducing the incidence of human campylobacteriosis because this pathogen can achieve stable and asymptomatic chicken colonization as commensal microbiota, thereby leading to bird-to-bird horizontal transmission after the invasion of farms (7). Despite the amount of research that has been performed on this subject, it is likely that the source and transmission routes of *C. jejuni* invasion in broiler farms have farm-to-farm variations (8). Comparatively, many attempts have been made to reduce this pathogen in the broilers during farm rearing using phage therapy (9), feed and/or water supplementation with short- or medium-chain fatty acids (FAs) (10–12), essential oils (13), organic acids (14), and probiotics (15), as practical control measures. These possible effectors are thought to have either direct or indirect bactericidal effects; however, further study would be required to clarify molecular basis of these approaches in terms of reducing *Campylobacter* in the field.

Chickens as a food source are largely divided into broiler chickens and laying hens. Broiler chickens are rapidly grown to slaughter weight, which is mainly affected by genetic background, digestive efficiency, and energy-use efficiency (16). During broiler chicken breeding, *C. jejuni* starts to colonize the gut at around 3–4 weeks of age, and then spread in the flocks, thereby becoming a burden at slaughter age (17–19). Similarly, in laying hens, which are used mainly for egg production, *C. jejuni* also exhibits increased colonization fitness between 0 and 4 weeks post-infection (*p.i*.) after experimental oral administration (20); moreover, both broilers and laying hens harbor *C. jejuni* at high percentages in the gut at slaughter age (21). Although laying hens are generally raised for longer periods compared with broiler chickens, the temporal dynamics of *C. jejuni* colonization in laying hens is not well-understood.

The recent advancements in the application of next-generation sequencing have allowed the investigation of the microbiome in specific organs of various animals. A recent study reported an association between *Campylobacter* burden and the microbiome in the cecum of broiler chickens (22); i.e., the authors reported a possible association between the decreased abundance of *Lactobacillus* spp. and high *Campylobacter* loads, raising questions pertaining to temporality and causation. In addition, Videnska and co-workers found different compositions of fecal microbiota between broiler chickens and laying hens at 30 or 61 weeks of age (23). However, they used chickens bred at different ages under different environments and using distinct feeds. It is likely that the fecal microbiota composition of laying hens varies according to their age; the gut microbiota of young hens are quite complex, whereas those of older hens are simpler and consist mainly of the phyla *Bacteroidetes* and *Firmicutes* (24). These observations suggest the alteration of the gut microbiota in laying hens during long-term growth, thus underscoring the need to monitor the temporal characteristics of gut microbiota in laying hens during breeding under similar environmental and feed conditions.

Based on this background, here we examined the dynamics of *C. jejuni* and microbiota compositions in the cecum of laying hens after experimental infection. After observing the time-to-time differences in the colonization fitness of *C. jejuni* and microbiota composition between 8 and 16 weeks *p.i*., we performed comparative lipidome analyses between these time points. Finally, we discussed their possible associations.

## Materials and Methods

### Bacterial Strain and Media

The *C. jejuni* 81-176 strain was employed as the inoculum in the chicken infection experiment. Bacteria were grown on Mueller-Hinton agar (MHA) or in Mueller-Hinton broth (MHB) (Merck, Darmstadt, Germany) at 42°C for 20 h under microaerophilic conditions using AnaeroPack-MicroAero system (Mitsubishi Gas Chemicals, Tokyo, Japan), unless otherwise indicated.

### Chicken Infection Experiment

Two-week-old female specific-pathogen-free (SPF) white leghorn (Line-M) chickens (*n* = 38 in total) were obtained from Nisseiken (Yamanashi, Japan) and introduced into our animal facility at the ABSL2 level. Animals were fed in sterilized cages *ad libitum* with sterile water and antibiotic-free pellet diets (CR, Nisseiken) at 25°C with lighting from 9 a.m. to 5 p.m. in biosafety level 2 room. To prepare the bacterial inoculum, *C. jejuni* 81-176 was microaerobically grown in MHB at 42°C for 20 h using AnaeroPack-Microaero (Mitsubishi Gas Chemicals, Tokyo, Japan). The bacterial culture was then washed twice with PBS, and adjusted to 6.84 log CFU per 1 ml of PBS. One milliliter aliquots of the bacterial suspensions was orally inoculated into each bird via 18G-feeding gavage (Thermo Fisher Scientific, Waltham, MA, USA). At 0, 2, 8, 16, 24, 32, and 40 weeks *p.i*., five each animals were sacrificed per time point and samples of at least 1 g of cecum content were aseptically collected. Simultaneously, whole blood was collected from animals at 2, 8, 16, and 24 weeks *p.i*. (two each birds per time point), followed by centrifugation at 3,000 rpm for 5 min, to collect sera. For the control, three animals were fed for 2 weeks after their introduction, and their cecal and serum samples were collected in a similar manner. The numbers of *C. jejuni* from the cecum samples were enumerated according to the method of ISO 10272-2: 2017 (25).

Experiments utilizing animals were approved by the board of Animal Welfare and Ethical Committee of the National Institute of Health Science with the approval number of 680.

### Enumeration of *C. jejuni* in Chicken Ceca

*Campylobacter jejuni* 81-176 was enumerated in chicken ceca essentially as described previously (20). Briefly, 1 g samples of fresh cecum were suspended in 9 ml of sterile buffered peptone water (BPW; Merck, Darmstadt, Germany); 1 ml aliquots of the BPW suspension and its serial dilutions were then spread on mCCDA agar plates (Oxoid, Hampshire, UK) and microaerobically incubated at 42°C for 48 h. The number of typical colonies was counted, and at least five suspected colonies per plate were subjected to real-time PCR to confirm *C. jejuni*, as described previously (26). Fisher's extract test was used to assess the statistical significance of the differences in the number of bacteria between the groups (2 and 8 vs. 16–40 weeks *p.i*.).

### DNA Extraction

Three representative chicken cecum samples were selected at each time point, to exclude the samples with maximum and minimum bacterial counts, and subjected to 16S rRNA sequencing analysis. Aliquots of the BPW suspensions (1 ml) were centrifuged at 21,500 × *g* for 10 min at 4°C. The pellets were then resuspended in 400 μl of homogenization solution containing 2 μl of proteinase K (Promega, Madison, WI, USA). After incubation at 37°C for 10 min, the samples were vortexed for 5 min with Zirconia beads (ZircoPrep Mini; Nippon Genetics, Tokyo, Japan) on a Disruptor Genie instrument (Scientific Industries, Bohemia, NY, USA). After centrifugation at 11,000 × *g* for 5 min, 100 μl of each supernatant were transferred into 300 μl of lysis buffer (Promega). DNA extraction was then carried out using a Maxwell Blood DNA kit in a Maxwell RSC instrument (Promega). The concentration and quality of the extracted DNA were measured on a Tape Station 4150 system (Agilent Technologies, Santa Clara, CA, USA), and the samples were stored at −80°C until use.

### 16S rRNA Gene Sequencing

Barcoded semi-conductor sequencing analysis was performed essentially as described previously (27). Briefly, the 16S rRNA V5–V6 region sequences were amplified from 2 to 4 ng of DNA from each sample by PCR using the primers 799f and 1115r (27). The PCR amplicons were purified using E-gel Size Select 2% (Thermo Fisher Scientific) and Agencourt AMPure XP magnetic beads (Beckman Coulter, Brea, CA). After measuring DNA concentration using the Ion library quantification kit (Thermo Fisher Scientific), equal quantities of tagged amplicons were pooled. The pooled DNA samples (5 pM per sample) were then subjected to the Ion Chef and Ion PGM (400 bases) sequencing platform using a 318v2 chip (Thermo Fisher Scientific), according to the manufacturer's instructions.

### Analysis of Microbiome Composition Data

FASTAQ files generated here were processed using the CLC Genomic Workbench ver. 20 (CLC-Qiagen, Aarhus, Denmark) to remove barcode sequences and low-quality sequences, which were defined as sequences with <275 bases, with ambiguous bases and homopolymers >6 bases, or without a barcode and a primer sequence. The 16S gene copy numbers were adjusted to 100,000 per a sample and taxonomical classification was carried out using the RDP pipeline (28) with an 80% confidence threshold. Operational taxonomy units (OTUs) were assigned using the average neighbor algorithm at 99% similarity on the RDP program, and the obtained OTUs which was then subjected to Permutational multivariate analysis of variance (PERMANOVA) test to calculate the statistical significance between three groups (group 1: 0 w *p.i*., group 2: 2 w and 8 w *p.i*., group 3: ≥16 w *p.i*.) by Bray-Curtis dissimilarity index under 10,000 times permutation using in-house program. Calculation of Shannon diversity indexes and Simpson indexes, and principle coordinate analysis (PCoA) were performed using Metagenome@KIN program (World Fusion, Tokyo, Japan) accordingly. All raw sequences were deposited into the DDBJ/GenBank database with accession number DRA009061 in BioProject PRJDB8861.

### Cytokine Assay

Semi-quantitative cytokine assays were performed using the RayBio® C-Series Gallus (Chicken) Cytokine Array C1 kit (Raybiotech, Peachtree Corners, GA, USA), according to the manufacturer's instructions. For this assay, two representative serum samples collected from laying hens at 2, 8, 16, and 24 weeks *p.i*. were used in duplicate sets. Chemiluminescence detection was performed using an ImageQuant LAS 500 system (Cytiva, Marlborough, MA, USA). Densitometrical data analyses were performed according to the guidelines of the manufacturer.

### Lipidome Analysis

#### Sample Preparation

Each pair of cecum samples collected at 8 weeks *p.i*. (samples 8-1 and 8-2), 16 weeks *p.i*. (16-1 and 16-2), and 24 weeks *p.i*. (24-1 and 24-2) (two samples per the time point) was subjected to a lipidome analysis. To extract lipids, 0.5 mg from each sample were sonicated in 300 μl of homogenization solution (CHCl_3_:methanol, = 1:2), followed by vortexing for 20 min at 20°C. The homogenate was then mixed with 20 μl of distilled water and vortexed again prior to centrifugation at 1,670 × *g* for 10 min at 20°C. The resultant supernatant was used as a sample in the subsequent analysis.

#### Analytical Equipment and Conditions

Three-microliter aliquots of the above-mentioned supernatants were injected for non-biased lipidome analysis using UPLC (Waters) in combination with Triple TOF 6600 (AB Sciex, Framingham, MA, USA) essentially as described previously (29). Mobile phase A consisted of 1:1:3 acetonitrile:methanol:water (v/v/v) with 5 mM ammonium acetate and 10 nM EDTA. Mobile phase B was 100% isopropanol with 5 mM ammonium acetate and 10 nM EDTA. The LC column was an Acquity UPLC Peptide BEH C18 column (50 × 2.1 mm; 1.7 μm; 130 Å). The gradient was 0 min, 0% B; 1 min, 0% B; 5 min, 40% B; 7.5 min, 64% B; 12.0 min, 64% B; 12.5 min, 82.5% B; 19 min, 85% B; 20 min, 95% B; 20.1 min, 0% B; and 25 min, 0% B. The column flow rate was 0.3 ml/min and the autosampler temperature was 5°C. The column temperature was 45°C. MS was performed on a TripleTOF 6600 system equipped with a DuoSpray ion source. All analyses were performed in the high sensitivity mode for both TOF–MS and product ion scanning. Data-dependent MS/MS acquisition (DDA) was used. The common parameters in both positive and negative ion mode were as follows: collision energy, 45 V; collision energy spread, 15 V; mass range, m/z 140–1,700; temperature, 300°C; and declustering potential, 80 V.

### Statistical Analysis

The statistical differences of bacterial numbers among the different age groups (2, 8, 16, 24, 32, and 40 weeks *p.i*.) were calculated by Steel-Dwass test, and *P* < 0.05 were considered to be significant. To compare 16S rRNA gene DNA sequence data between 2/8 and 16/24/32/40 weeks *p.i*., relative abundances were comparatively analyzed by a non-parametric joint ranked Dunn test, and *P* < 0.05 were considered to be significant. The MS/MS spectra of each fragment ranged from 70 to 1,700 m/z obtained by lipidome analyses were analyzed using the MS-DIAL program and MS-FINDER software (30) to identify and classify lipids. The statistical significance of the differences among the different age groups (8, 16, and 24 weeks *p.i*.) was calculated by Bonferroni test and *P*-value of <0.05 were considered to be significant.

## Results

### Colonization Fitness of *C. jejuni* in the Cecum of Laying Hens Over a Period of 40 Weeks

**Figure 1:**
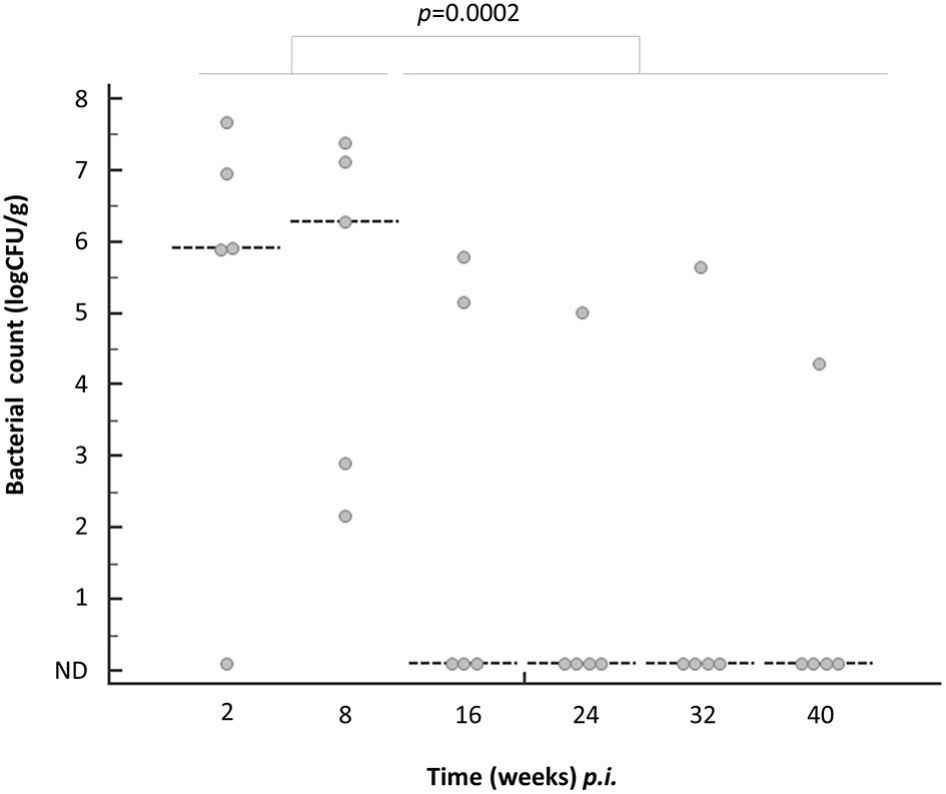
Temporal colonization fitness of *C. jejuni* 81-176 in the caecum of laying hens. Each dot (gray) represents bacterial numbers detected per a gram of caecum. Dotted line represents median at each sampling time point. Statistical significance of the differences in the number of bacteria between the groups (2 and 8 weeks *p.i*. vs. 16–40 weeks *p.i*.) by Fisher's extract test.

After oral infection with *C. jejuni* 81-176, the inocula were stably recovered from the cecum of laying hens for up to 8 weeks *p.i*.; the number of pathogens recovered was 7.08 and 6.90 log CFU/g at 2 and 8 weeks *p.i*., respectively (Figure 1). At 16 weeks *p.i., C. jejuni* were recovered only from two chickens (40%, 2/5 birds), with average means of 5.18 log CFU/g (Figure 1). At 24, 32, and 40 weeks *p.i., C. jejuni* was recovered from one out of five birds, at 5.02, 5.65, and 4.30 log CFU/g, respectively (Figure 1). Statistically, there was a significant difference (*P* = 0.0002) in the recovered bacterial burden between 2 and 8 weeks *p.i*. (defined as *C. jejuni* colonizer) and 16–40 weeks *p.i*. (*C. jejuni* excluser) (Figure 1). Thus, these data indicate that *C. jejuni* retained colonization at an early stage, but tended to have a reduced colonization fitness after 16 weeks *p.i*. in the gut of laying hens.

### Alteration of the Cecum Microbiome of the Laying Hens

After confirming the alteration in the colonization fitness of *C. jejuni* in the cecum of the SPF laying hens, their bacterial community structures (*n* = 3 each at 0, 2, 8, 16, 24, 32, and 40 weeks *p.i*.) were analyzed using a 16S rRNA gene sequencing approach. The Ion Torrent sequencer output 217,473–333,238 reads, and after filtering, 160,209–235,614 reads were remained (Supplementary Table 1). After normalization to 100,000 valid reads per a sample, a total of 446, 160, 76, 43, and 27 taxa were finally detected at the genus, family, order, class, and phylum levels, respectively, by RDP program. Shannon diversity index showed the increased trend at >16 weeks *p.i*. (Supplementary Table 1). In contrast, Simpson index resulted in the decreased trends in the means at >16 weeks *p.i*. (Supplementary Table 1). Permutational multivariate analysis of variance analysis showed the significant differences of the bacterial community between three groups (group 1: 0 w *p.i*., group 2: 2 and 8 w *p.i*., group 3: ≥16 w *p.i*.) at *R*^2^ of 0.375 and *P*-value of 0.0001.

#### Phylum Level Comparison

**Figure 2:**
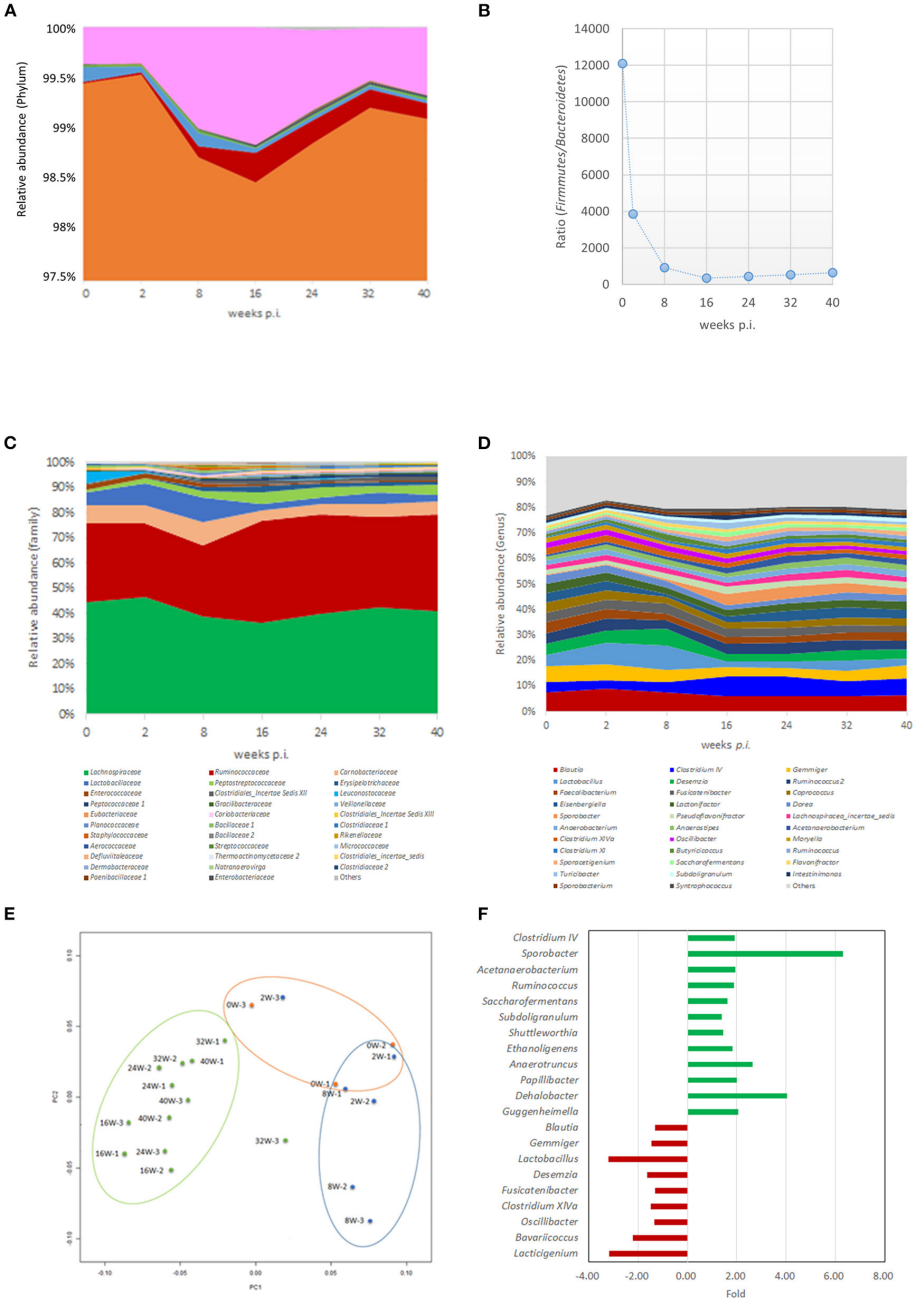
Altered microbiome in the caecum of laying hens during infection experiments. **(A)** Time-dependent dynamics of representative bacterial phylum as detected in caecal samples of laying hens. **(B)** Time-dependent dynamics of the *Firmicutes*/*Bacteroidetes* (F/B) ratio of the caecal microbiomes. **(C)** Time-dependent *d*ynamics of representative bacterial family as detected in caecal samples of laying hens. **(D)** Time-dependent dynamics of representative bacterial genera as detected in caecal samples of laying hens. **(E)** Principle coordinate analysis (PCoA) plot of representative bacterial phylum as detected in caecal samples of laying hens. Dots are shown in different colors by time points (0 weeks *p.i*., orange; 2 weeks and 8 weeks *p.i*., blue; >16 weeks *p.i*., green). **(F)** Fold changes in the relative abundances of representative bacterial genera between >16 weeks *p.i*. and <8 weeks *p.i*. Bacterial genera exhibiting the increased relative abundance at >16 weeks *p.i*. compared with 2/8 weeks *p.i*. are shown with green color, and those exhibiting the decreased relative abundances at >16 weeks *p.i*. compared with 2/8 weeks *p.i*. are shown with red color, respectively.

Overall, the main bacterial phyla detected in the cecum of laying hens were represented by *Firmicutes*, followed by *Actinobacteria, Bacteroidetes*, and *Proteobacteria*, with means ± *SD* of 99.05 ± 0.65, 0.69 ± 0.59, 0.14 ± 0.14, and 0.07 ± 0.07%, respectively (Figure 2A). Compared with 0 weeks *p.i*., the results obtained at >16 weeks *p.i*. showed significant differences in the relative abundance of *Firmicutes* (z = −2.36, *P* = 0.036) and *Bacteroidetes* (z = 2.96, *P* = 0.006), while the abundance of *C. jejuni* colonizers was not significantly different (Figure 2A), as assessed using the non-parametric Dunn test. The *Firmicutes*/*Bacteroidetes* (F/B) ratio, which is related to age in humans (31), was decreased in a time-dependent manner, from 0 weeks *p.i*. (mean, 12,104) to 16 weeks *p.i*. (340), and gradually increased thereafter (mean = 439, 535, and 648 at 24, 32, and 40 weeks *p.i*., respectively) (Figure 2B).

#### Family-Level Comparison

Throughout the experimental periods, the family *Lachnospiraceae* was predominant (41.20 ± 3.76%), followed by *Ruminococcaceae* (34.63 ± 4.99%), and *Carnobacteriaceae* (4.80 ± 3.77%) (Figure 2C and Supplementary Figure 1). According to time point, the family *Lachnospiraceae* showed a temporal decrease in relative abundance from 0 to 16 weeks *p.i*.; in turn, it increased thereafter, up to 32 weeks *p.i*. (Figure 2C and Supplementary Figure 1). The families *Ruminococcaceae* and *Erysipelotrichaceae* showed an increased relative abundance at 16 weeks *p.i*., as a plateau, thereby stably existing in these samples (Figure 2C and Supplementary Figure 1). In contrast, the families *Carnobacteriaceae* and *Lactobacillaceae* exhibited an initial (up to 8 weeks *p.i*.) increase in their relative abundance, to then decrease after 16 weeks *p.i*. (Figure 2C and Supplementary Figure 1). Finally, the families *Peptostreptococcaceae* and *Clostridiales Incertae Sedis* XII showed a time-dependent increase in relative abundance (Figure 2C and Supplementary Figure 1), whereas the family *Enterococcaceae* showed a time-dependent decrease in relative abundance (Figure 2C and Supplementary Figure 1).

#### Genus-Level Comparison

Among all tested samples, the genus *Blautia* was predominant (7.08 ± 1.16%), followed by *Clostridium* IV (5.57 ± 1.85%), *Lactobacillus* (4.80 ± 3.77%), and *Gemmiger* (4.72 ± 1.42%) (Table 1, Figure 2D). According to sampling time point, before *C. jejuni* infection (0 weeks *p.i*.), *Blautia* was the predominant genus (7.65 ± 0.13%), followed by *Gemmiger* (6.46 ± 1.10%), *Weissella* (4.63 ± 1.34%), and *Faecalibacterium* (4.52 ± 0.73%) (Table 1, Figure 2D, and Supplementary Figure 2). At 2 and 8 weeks *p.i*., the predominant genera were *Lactobacillus* (8.98 ± 4.25%), *Blautia* (8.30 ± 1.01%), *Desemzia* (5.76 ± 1.30%), and *Gemmiger* (5.57 ± 1.21%) (Table 1, Figure 2D, and Supplementary Figure 2). At >16 weeks *p.i., Clostridium* IV was the predominant genus (6.96 ± 1.04%), followed by *Blautia* (6.33 ± 0.68%), *Ruminococcus* (3.88 ± 0.69%), and *Gemmiger* (3.86 ± 0.87%) (Table 1, Figure 2D, and Supplementary Figure 2).

**Table 1:** Relative abundance of bacterial genera (%) in the cecum of laying hens.

**Genus**	**0 weeks *p.i*.**	**2/8 weeks** ***p.i***.		**16/24/32/40 weeks** ***p.i***.		
	**Mean ±*SD* (%)**	**Mean ±*SD* (%)**	***P-value* to**	**Mean ±*SD* (%)**	***P-value*** to ^*^	
			**0 w *p.i*.**		**0 w *p.i*.**	**2/8 w *p.i*.**
**INCREASED RELATIVE ABUNDANCE AT** **>16 WEEKS** ***P.I***. **COMPARED WITH 2/8 WEEKS** ***P.I***.						
*Clostridium* IV	3.91 ± 0.80	3.61 ± 0.37	1.0000	6.96 ± 1.04	**0.0488**	**0.0017**
*Sporobacter*	0.49 ± 0.35	0.58 ± 0.40	1.0000	3.66 ± 0.96	**0.0239**	**0.0030**
*Acetanaerobacterium*	0.83 ± 0.18	1.22 ± 0.26	1.0000	2.37 ± 0.40	**0.0036**	**0.0107**
*Ruminococcus*	0.89 ± 0.16	0.86 ± 0.13	1.0000	1.62 ± 0.21	**0.0488**	**0.0017**
*Saccharofermentans*	0.79 ± 0.13	0.89 ± 0.23	1.0000	1.44 ± 0.34	**0.0255**	**0.0138**
*Subdoligranulum*	1.08 ± 0.19	0.91 ± 0.17	0.8886	1.27 ± 0.25	0.9690	**0.0127**
*Shuttleworthia*	0.64 ± 0.09	0.76 ± 0.12	1.0000	1.11 ± 0.21	**0.0085**	**0.0208**
*Ethanoligenens*	0.53 ± 0.16	0.62 ± 0.16	1.0000	1.14 ± 0.35	**0.0225**	**0.0265**
*Anaerotruncus*	0.41 ± 0.08	0.39 ± 0.10	1.0000	1.03 ± 0.14	**0.0488**	**0.0017**
*Papillibacter*	0.40 ± 0.18	0.42 ± 0.06	1.0000	0.84 ± 0.17	**0.0144**	**0.0044**
*Dehalobacter*	0.16 ± 0.03	0.21 ± 0.08	1.0000	0.85 ± 0.29	**0.0085**	**0.0063**
*Guggenheimella*	0.15 ± 0.02	0.37 ± 0.31	1.0000	0.76 ± 0.13	**0.0344**	**0.0452**
**DECREASED RELATIVE ABUNDANCE AT** **>16 WEEKS** ***P.I***. **COMPARED WITH 2/8 WEEKS** ***P.I***.						
*Blautia*	7.65 ± 0.13	8.30 ± 1.01	1.0000	6.33 ± 0.68	0.0989	**0.0048**
*Lactobacillus*	4.37 ± 3.03	8.98 ± 4.25	0.5700	2.81 ± 1.43	1.0000	**0.0226**
*Gemmiger*	6.46 ± 1.10	5.57 ± 1.21	1.0000	3.86 ± 0.87	**0.0324**	**0.0361**
*Desemzia*	4.19 ± 2.19	5.76 ± 1.30	0.4308	3.54 ± 1.16	1.0000	**0.0265**
*Fusicatenibacter*	3.60 ± 0.39	3.78 ± 0.54	1.0000	2.89 ± 0.41	0.1791	**0.0095**
*Clostridium XlVa*	2.31 ± 0.40	2.39 ± 0.39	1.0000	1.63 ± 0.24	0.0939	**0.0036**
*Oscillibacter*	2.30 ± 0.66	2.18 ± 0.34	1.0000	1.64 ± 0.26	0.1408	**0.0286**
*Bavariicoccus*	1.24 ± 0.30	1.00 ± 0.16	1.0000	0.45 ± 0.08	**0.0111**	**0.0053**
*Lacticigenium*	1.24 ± 0.32	0.79 ± 0.26	1.0000	0.25 ± 0.06	**0.0085**	**0.0063**

*Bacterial genera exhibiting >0.50% of relative abundance on average in total are listed. p.i., post-infection*.

* *P-values were calculated using a non-parametric joint ranked Dunn test*.

*Bold values represent P-values of <0.05*.

#### Characterization of the Time-Dependent Dynamics of Cecal Microbiota

The principal coordinate analysis illustrated a distinct distribution of the samples at >16 weeks *p.i*. compared with those observed at 0, 2, and 8 weeks *p.i*. (Figure 2E). A comparison of the relative abundance of each bacterial genus between the groups (2 and 8 vs. >16 weeks *p.i*.) revealed a significant alteration in the relative abundance of several bacterial genera: a total of 12 and 9 genera exhibited a significant increase or decrease in their relative abundance between the groups, respectively (Table 1, Figure 2F). The 12 genera that showed an increased relative abundance at >16 weeks *p.i*. were *Clostridium* IV, *Sporobacter* (family *Clostridiaceae*), *Acetanaerobacterium, Ruminococcus, Subdoligranulum, Ethanoligenens, Anaerotruncus, Papillibacter* (family *Ruminococcaceae*), *Shuttleworthia* (family *Lachnospiraceae*), *Dehalobacter* (family *Peptococcaceae*), and *Guggenheimella* (family *Clostridiales incertae sedis*), all of which were classified in the order *Clostridiales* (Table 1, Figure 2F). In contrast, among the nine genera exhibiting a time-dependent decrease in relative abundance, five genera were in the order *Clostridiales* (*Blautia, Gemmiger, Fusicatenibacter, Clostridium* XIVa, and *Oscillibacter*), whereas the remaining genera (*Lactobacillus, Desemzia, Bavariicoccus*, and *Lacticigenium*) were in the order *Lactobacillales* (Figure 2F and Supplementary Figure 2). Thus, these data clearly suggest time-dependent alterations of the chicken gut microbiota composition throughout the experimental period.

### Time-Course Dynamics of Cytokine Production in the Serum of Laying Hens

**Figure 3:**
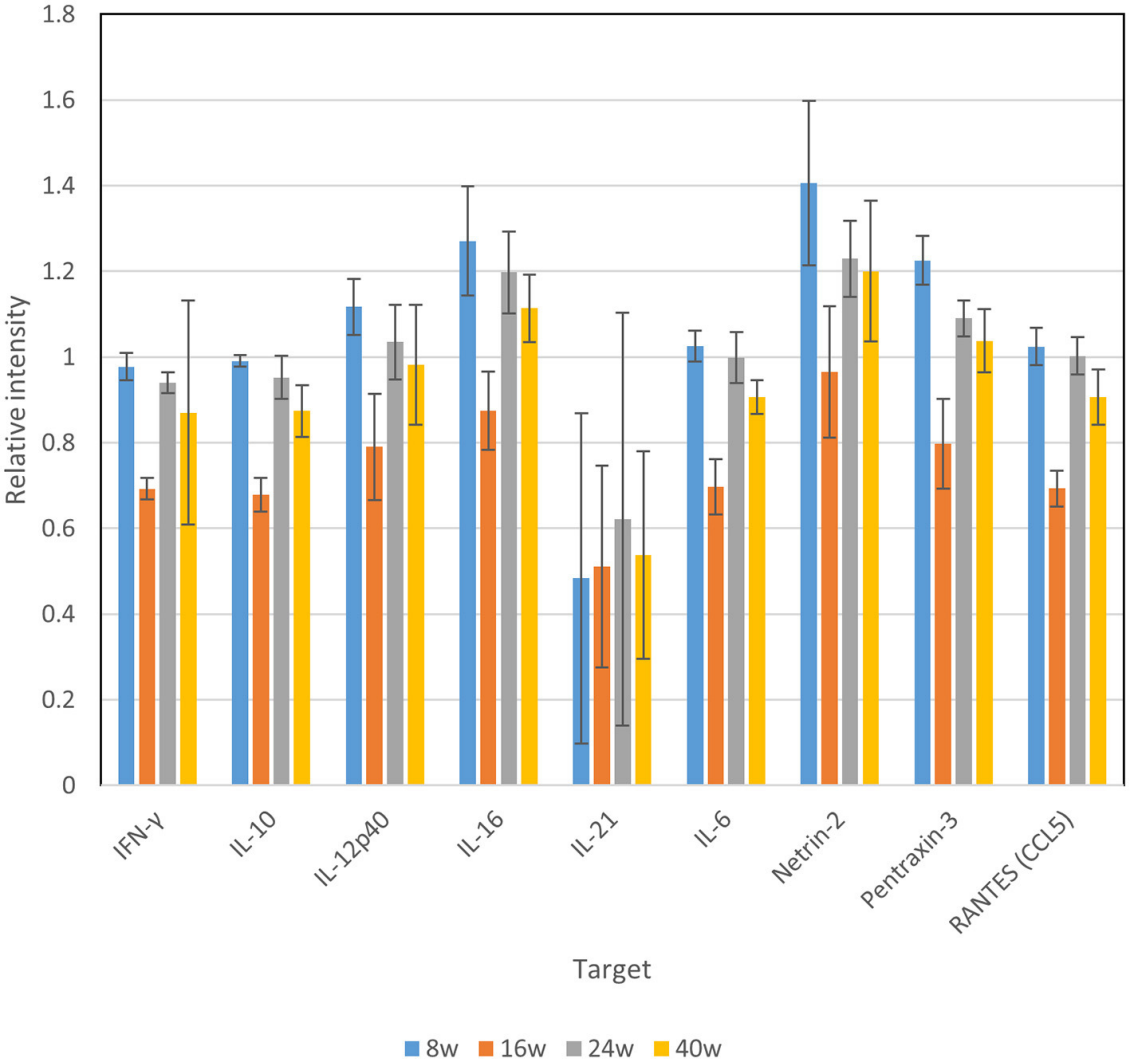
Cytokine production in the sera of laying hens after experimental *C. jejuni* infection.

The levels of representative chicken cytokines [IFN-γ, IL-10, IL-12p40, IL-16, IL-21, IL-6, netlin-2, pentraxin-3 (PTX-3), and RANTES (CCL5)] in the serum samples were comparatively examined using a semi-quantitative membrane array. Compared with the serum samples collected at 8 weeks *p.i*., the samples obtained at 24 and 40 weeks *p.i*. exhibited no apparent differences in all target molecules (Figure 3). In contrast, the samples collected at 16 weeks *p.i*. exhibited a reduced level of cytokines, with the exception of IL-21 (Figure 3). Thus, these data indicate the presence of altered cytokine production at 16 weeks *p.i*. compared with the other time points of the experimental period.

### Lipid Metabolic Profiles in the Cecum of Laying Hens

Untargeted LC-MS/MS analyses were conducted to comparatively measure chick cecum lipids and lipid metabolites in a total of six cecum samples collected at 8, 16, and 24 weeks *p.i*. These comparative analyses revealed that 22 or 36 lipids were significantly increased or decreased in the samples collected at 16/24 weeks *p.i*., respectively, compared with those obtained at 8 weeks *p.i*. (Table 2 and Supplementary Figure 2). These dynamics between the time courses were explained as follows.

**Table 2:** Summary of altered lipids in the gut of laying hens between 8, 16, and 24 weeks *p.i*.

**Category**	Lipid component^1*^	**Average**				***P*****-value**^**1,2**^		
		**Blank**	**8 w**	**16 w**	**24 w**	**8 w vs. 16 w**	**8 w vs. 24 w**	**16 w vs. 24 w**
**INCREASED AT 16 WEEKS AND/OR 24 WEEKS** ***P.I***. **(16 W/24 W) COMPARED WITH 8 WEEKS** ***P.I***. **(8 W)**								
Fatty acid metabolite	FA (27:1)	21	2,905	11,031	12,297.5	0.123	**0.003**	0.594
	FA (28:0)	64	1,220.5	4,682.5	4,289.5	**0.012**	**0.026**	0.186
	AAHFA (5:0/26:0)	59	560.5	2,532.5	4,867	0.426	**0.032**	0.371
	AH2FA (4:0/25:0)	22	713.5	1,605	2,449	0.411	**0.036**	0.432
	CmpE (26:0)	53	335.5	1,167	1,462	0.249	**0.010**	0.549
Glycerolipid	TG (45:1)	203	336.5	800	798.5	0.370	**0.049**	0.997
	MGDG (18:0/20:2)	11	439	1,652.5	596	**0.041**	0.316	**0.044**
Phospholipid	MLCL (16:0/16:0/18:2)	0	237	892.5	452.5	**0.006**	0.069	**0.038**
	CL (e/e)(28:1e/30:1e)	11	2,024.5	2,591.5	5,378.5	0.568	**0.038**	0.099
	CL (e/e)(30:1e/33:3e)	0	1,178.5	1,298.5	2,452.5	0.835	**0.024**	0.222
	CL (30:1e/30:3e)	0	125	219	296.5	0.140	**0.013**	0.198
	DLCL (16:0/16:0)	16	3,637.5	17,440	10,366	0.175	**0.045**	0.327
	DLCL (16:0/18:2)	0	1,853	5,436	2,487	**0.002**	0.628	0.200
	HBMP (18:0/18:1/g18:1)	11	100	237	331.5	**0.017**	0.240	0.496
	HBMP (14:0/16:0/18:0)	0	158.5	341.5	424.5	0.081	**0.047**	0.289
	PG (18:2/18:2)	16	1,353.5	2,083.5	2,179	**0.007**	0.620	0.950
Sphingolipid	CerPI (d44:0)	0	158	700.5	990	**0.056**	**0.039**	0.060
	AcylCerPG (t18:0/16:0/15:0)	0	361	2,758.5	2,101	0.052	**0.041**	0.324
Sterol lipids	StE (18:1)	930	50,481.5	79,812.5	118,907.5	0.286	**0.030**	0.200
	StE (18:0)	236	4,940	9,740.5	11,280	0.298	**0.007**	0.646
	SISL G1	157	30,164.5	64,382	49,213	**0.049**	0.067	0.181
	Stigmasterol G1	91	5,295	8,517	7,433	0.275	**0.047**	0.603
**DECREASED AT 16 WEEKS AND/OR 24 WEEKS** ***P.I***. **(16 W/24 W) COMPARED WITH 8 WEEKS** ***P.I***. **(8 W)**								
Fatty acid metabolite	FA (17:0)	2,265	1,335,104	909,822.5	368,852.5	0.619	**0.038**	0.543
	FA (18:0)	151,628	6,089,247	4,739,226.5	2,362,394.5	0.711	**0.036**	0.546
	FA (19:1)	0	555,886	95,071.5	64,656.5	0.018	**0.004**	0.246
	FA (20:3)	23	10,567.5	2,823.5	2,095	0.123	**0.039**	0.717
	FA (24:1)	348	246,110.5	127,115	94,823.5	0.088	**0.009**	0.309
	FA (24:2)	35	21,430	13,737.5	12,986	0.298	**0.044**	0.887
	FA(O) (18:0)	67	93,970	20,635	16,832	0.067	**0.043**	0.862
	FA(aOH)(16:0)	164	1,059,824.5	315,401	86,486	0.173	0.135	**0.034**
	FA(aOH)(18:0)	241	270,228	175,671	65,140.5	0.098	**0.025**	**0.020**
	FA(aOH)(19:0)	40	29,623.5	23,000	12,582.5	0.120	**0.020**	0.059
	FA(aOH)(22:1)	21	35,900.5	22,324.5	20,211.5	0.061	**0.015**	0.252
	FA(aOH)(24:1)	66	389,587.5	212,609.5	181,281	0.060	**0.046**	0.515
Glycerolipid	MGDG(sn2+O)(16:0/16:0)	44	24,594.5	8,656.5	11,126	**0.018**	**0.033**	0.301
	AMGDGM(e) (16:1e/17:0/18:1)	0	1,169.5	467	344.5	**0.037**	**0.045**	0.146
	AMGDG(e)(18:1e/14:0/18:1)	0	972.5	538.5	417.5	**0.043**	**0.020**	0.244
	GPMGDG(e)(34:1e)	34	23,957	11,863.5	10,900	0.189	**0.044**	0.870
	GPMGDG(e)(34:3e)	75	5,551	3,790	4,866	**0.041**	0.768	0.655
	DGDG(16:0/18:2)	21	1,161	1,267	728.5	0.705	**0.023**	0.228
	DGDG(18:0/18:1)	0	643.5	442	308	0.421	**0.018**	0.554
	DGDG(e)(18:1e/18:1)	0	6,650	2,918	1,610	0.244	**0.015**	0.563
	DGDG(e)(17:1e/18:1)	0	712.5	337.5	170.5	0.172	**0.025**	0.391
	ADGDG(16:0/18:1/g16:0)	0	746.5	630	415.5	0.457	**0.048**	0.255
	TG(51:4)	51	3,101	4,343.5	1,829	0.702	**0.037**	0.492
	TG(60:4)	0	1,959	1,898.5	1,046	0.934	**0.035**	0.372
	DG (16:0/19:1)	338	68,852.5	38,753.5	31,749	**0.025**	0.117	0.571
Phospholipid	PE(16:0/16:1)	11	364.5	262.5	765.5	**0.016**	0.531	0.459
	Lyso PC (20:0)	190	2,531.5	2,373.5	427	0.893	**0.014**	0.284
	PE (18:0)	134	1,619.5	1,485	373	0.844	**0.014**	0.286
Sphingolipid	AcylCer(phyto_aOH)(t18:1/24:0/2:0)	0	4,412.5	2,732	708.5	0.281	**0.010**	0.226
	3-O-AcylSM (d36:1/16:0)	0	579	427	188	0.210	0.082	**0.006**
Sterol lipid	Taurin (19:1)	16	962.5	532.5	157.5	**0.049**	**0.041**	0.133
	dehydro cholesterol	856	2,425.5	3,853.5	1,408.5	0.212	**0.007**	0.127
Prenol lipid	Coenzyme Q9H2	155	78,620.5	11,985.5	14,275.5	**0.049**	**0.031**	0.747
	Coenzyme Q11	88	17,740	7,237.5	3,685	0.089	**0.014**	0.299
	Coenzyme Q11H2	0	3,928.5	952.5	757.5	0.077	**0.035**	0.805
Others	Tocopherol	76	16,937	6,859.5	5,661.5	**0.003**	**0.040**	0.459

*p.i., post-infection*.

*1 The abbreviation of each lipid component is defined in Supplementary Table 2.

*2 P < 0.05, indicated in bold.

### Fatty Acids

Fatty acids (FAs), such as 27:1 and 28:0, as well as the FA ester of hydroxy fatty acid (AH2FA) 4:0/25:0 and acyl α-hydroxy fatty acid (AAHFA) 5:0/26:0, the latter of which was recently identified as a gut-microbiota-specific lipid (30), were present at higher levels at 24 weeks *p.i*. compared with 8 weeks *p.i*. (Table 2). In contrast, FAs 17:0, 18:0, 19:1, 20:3, 24:1, and 24:2, as well as α-hydroxy fatty acids [FA(aOH)] (18:0, 19:0, 24:1, 22:1, and 19:0), and FA(O) 18:0, showed decreased levels in the samples collected at 16 and/or 24 weeks *p.i*. compared with those obtained at 8 weeks *p.i*. (Table 2). Moreover, FAs 16:0 and 18:0 showed a continued decrease at 24 weeks *p.i*. compared with 16 weeks *p.i*. (Table 2).

### Glycerolipids

Among the glycerolipids, triacylglycerolipid (TG) (C45:1), and monoglycosyl diacylglycerol (MGDG) (18:0/20:2) were increased at 24 or 16 weeks *p.i*. compared with 8 weeks *p.i*., although the increase of the latter was temporally detected only at 16 weeks *p.i*. (Table 2). Other glycerolipids, such as monogalactosyldiacylglycerol (MGDG) (sn2+O) (16:0/16:0), acylmonoglycosyl diacylglycerol (AMGDG) (16:1e/17:0/18:1), AMGDG (18:1e/14:0/18:1), glycerophosphomonoglycosyl monoacylglycerol (GPMGDG) (34:1e), GPMGDG (34:3e), diglycosyldiacylglycerol (DGDG) (16:0/18:2), DGDG (18:0/18:1), diglycosyl 1-alkyl, 2-acylglycerol [DGDG(e)] (18:1e/18:1), DGDG(e) (17:1e/18:1), TG (51:4), TG (60:4), and diacylglycerol (DG) (16:0/19:1), were decreased at 16 and/or 24 weeks *p.i*. compared with 8 weeks *p.i*. (Table 2).

### Phospholipids

Among the phospholipids, monolysocardiolipin (MLCL) (16:0/16:0/18:2), cardiopin (CL) (28:1e/30:1e), CL (30:1e/33:3e), CL (30:1e/30:3e), dilysocardiolipin (DLCL) (16:0/16:0), DLCL (16:0/18:2), hemi bis (monoacylglycero) phosphate (HBMP) (18:0/18:1/g18:1), HBMP (14:0/16:0/18:0), and phosphatidyl glycerol (PG) (18:2/18:2) were increased at 16 or 24 weeks *p.i*. compared with 8 weeks *p.i*. (Table 2). Conversely, phosphatidyl ethanolamine (PE) (14:0/16:0/18:0), monoacylglycerophosphocholine (LysoPC) (20:0), and diacylglycerophosphoethanol (PE) (18:0) were decreased in the samples at 16 or 24 weeks *p.i*. compared with 8 weeks *p.i*. (Table 2).

### Sphingolipids

Phytoceramide and acyl phosphoglycerol were increased at 24 weeks *p.i*. compared with 8 weeks *p.i*. (Table 2). In turn, acyl ceramide (AcylCer) (phyto_aOH) (t18:1/24:0/2:0) and *N*-palmitoyl-d-erythro-sphingosylphosphorylcholine were decreased at 24 weeks *p.i*. compared with 8 weeks *p.i*. (Table 2).

### Sterol Lipids, Prenol Lipids, and Others

Sterol ester (StE) (18:0), StE (18:1), sitosterol (SISL) G1, and stigmasterol G1 were increased in the samples at 16 or 24 weeks *p.i*. compared with 8 weeks *p.i*. (Table 2). Conversely, 2-amino ethanesulfonic acid, taurine (19:1), and dehydro cholesterol were decreased in the samples at 16 and/or 24 weeks *p.i*. compared with 8 weeks *p.i*. (Table 2). Among the prenol lipids, coenzymes Q9H2, Q11, and Q11H2 exhibited decreased levels at 16 and/or 24 weeks *p.i*. compared with 8 weeks *p.i*. (Table 2). Finally, tocopherol was decreased at and 24 weeks *p.i*. compared with 8 weeks *p.i*. (Table 2).

## Discussion

The current study investigated the temporal colonization fitness of *C. jejuni* in the cecum of laying hens after experimental infection. In parallel with the decreased bacterial colonization fitness observed after 16 weeks *p.i*., compositional changes in the gut microbiota and lipids were observed, suggesting their possible correlations.

After invasion, *C. jejuni* initializes and prolongs gut colonization in the gut of broiler chicken up to the slaughter age (generally <8 weeks) (17–21). At <2 weeks of age, this pathogen is rarely detected in commercial chicken flocks, regardless of the production system (32, 33), which implies that a biological mechanism to resist colonization may be present in young chicks. As a possible explanation for this phenomenon, maternal antibodies might be partly responsible for the absence of *Campylobacter* in young chicks (34).

It is noteworthy that *C. jejuni* could maintain the colonization for up to 8 weeks *p.i*., which is the general time point for the slaughter of broiler chickens; however, *C. jejuni* exhibited a decrease in its colonization ability thereafter and up to 40 weeks *p.i*. As the feed and water supplied in this study contained no antibiotics and no compositional changes, it could be considered that such a decreased colonization ability might be triggered by the maturation of the host immune response or certain interactions with gut microbiota occurring during the experimental period. Further studies would be required to clarify that all laying hens might exhibit similar trends for *Campylobacter* colonization, throughout the quantitative detection of this pathogen.

Our data revealed a temporal decrease in the production of IFN-γ, IL-10, IL-12p40, IL-16, IL-6, netrin-2, PTX-3, and RANTES (CCL5) at 16 weeks *p.i*., and constant production of IL-21 in the serum. This host immune response is considered to be one of the imperative factors affecting *C. jejuni* colonization, although it remains controversial; Pielsticker et al. reported that triggering an innate and acquired immune response, especially in the very early phase, affected bacterial colonization (35). However, in most experimental studies, contradictory data regarding the immune response in chickens following *C. jejuni* colonization were reported; one study contended that the chicken immune system is inefficiently activated, which might contribute to the persistent colonization of *C. jejuni* in the chicken gut (36, 37). In contrast, another study showed the presence of an inflammatory response following *Campylobacter* infection in chickens (38). The occurrence of such immune responses upon *C. jejuni* colonization might be due to the supposed genetic heterogeneity of both the chicken hosts and *C. jejuni* (39). Our data suggest that the laying hens used in this study might not represent an animal with a significant immunomodulatory response against *C. jejuni* infection during long-term grow-out. It remains unknown why the laying hens showed temporal decreases in the production of most cytokines at 16 weeks *p.i*. It is possible that, at this stage, certain physiological shifts occur in laying hens, as reflected in the visual observation of coloring of combs and male–female discrimination (data not shown). In contrast, IL-21, which is a T-cell-derived cytokine that modulates T cell, B cell, and natural killer cell responses and regulates the Th17/Treg balance in mice (40), exhibited no clear alteration at 16 weeks *p.i*. Further studies are required to evaluate the possible role of T-cell-mediated immunity in the colonization of *C. jejuni* in laying hens.

Gut microbiota play a pivotal role in conferring resistance to, or promoting, infection by pathogenic microorganisms (41). In fact, the administration of a large dose of streptomycin disrupted normal gut microbiota, thereby increasing susceptibility to *Salmonella* infection (42). Similarly, germ-free chickens were more susceptible to *C. jejuni* colonization compared with chickens possessing conventional intestinal microbiota (43). *Campylobacter jejuni* is likely to cooperate and compete with diverse commensal microbiota, thus becoming part of a well-balanced gut microbial community (44). Johansen and colleagues also found that *C. jejuni* colonization affected the development and complexity of the microbial communities in the ceca of chicken up to 17 days of age (45). A more recent study revealed that the experimental inoculation of *C. jejuni* into 1-day-old broiler chicks modulated the cecal microbial community structure, with a higher abundance of *Firmicutes* at the expense of the phylum *Bacteroidetes* and other taxa at 3–4 weeks *p.i*. (46). Accordingly, our data also showed that the phylum *Firmicutes* predominated at 0 weeks *p.i*. (2 weeks age), but was replaced thereafter with the representatives of *Bacteroidetes* at 8–16 weeks *p.i*. (10–18 weeks of age). At >16 weeks *p.i*., among the phylum *Bacteroidetes*, the genus *Blautia* showed negative associations with *C. jejuni* colonization. Including the genus *Blautia*, all genera in the phylum *Bacteroidetes* are likely to express enzymes for the biosynthesis of propionate, one of the main short-chain fatty acids (SCFAs) in the chicken cecum (47), which suggests a possible alteration of lipid metabolism in the cecum of laying hens during the experimental period. Referring to a recent study that demonstrated the age-dependent dynamics of cecal microbiota in laying hens (24), our data provided the idea that experimental infection with *C. jejuni* might not affect the age-dependent dynamics of cecal microbiota composition drastically during the experimental period, whereas age-dependent shifts in the gut microbiota might affect the *C. jejuni* colonization properties. To clarify this issue, our future study would be performed to include unchallenged control groups at different ages, in same animal lot.

Regarding the bacterium-to-bacterium interplay, a positive correlation between the relative abundance of the genus *Clostridium* and *C. jejuni* colonization in the gut of broiler chickens has been reported (48). This might be due to the fact that *C. jejuni* acts as a hydrogen sink, thus leading to improved growth conditions for some *Clostridia* through increased fermentation (49) and organic acid production, which can be used by *C. jejuni* as an energy source. As a consequence, *C. jejuni* infection affects the metabolic end products derived from the intestinal microbiota of chickens. In support of this notion, a recent study showed that butyrate, one of the SCFAs that are biosynthesized by a series of *Clostridium* species (50), is directly sensed by *C. jejuni* through the BumSR two-component signal transduction system (51).

It is likely that gut microbiota affect intestinal lipid metabolism, including microbiota-dependent changes in bile acid metabolism (52). To obtain further information on the altered microbiota dynamics and *C. jejuni* colonization fitness, we performed comparative lipidome analyses using samples collected at three different time points (8, 16, and 24 weeks *p.i*.).

Among the elevated lipids at >16 weeks *p.i*., we found increased levels of phytosterols, such as stigmasterol and sitosterol, which can reduce the reabsorption of bile acids and cholesterol in the gut, thereby increasing fecal lipid levels (53), at 16 weeks *p.i*. compared with 8 weeks *p.i*. Considering that bile acids are steroid acids that are synthesized in the liver and then conjugated with a taurine residue to give anions called bile salts (54), our data demonstrating the decreased levels of sterol lipids (i.e., taurine and dehydro cholesterol) and sphingolipids (i.e., phytoceramide), which are components of bile acids (55, 56), at 16/24 weeks *p.i*. compared with 8 weeks *p.i*. suggest that bile acid reabsorption might be altered at these time points. In cecal digesta of goats that were fed a high-grain diet, the level of stigmasterol was negatively correlated with the abundance of the genus *Clostridium, Turicibacter*, SMB53, and *Pseudoramibacter* (41). Together with our microbiome data, potential negative associations between phytosterols and *Clostridium*/*C. jejuni* colonization in laying hens should be considered. The temporal quantification of bile acids in the gut and gallbladder would clarify the kinetics of bile acid synthesis and absorption and provide a link with their impact on gut microbiota in a future study.

Among the glycerolipids, the cecum samples collected at 16/24 weeks *p.i*. showed increased levels of TG (45:1) and MGDG (18:0/20:2), while an additional 13 glycerolipids were decreased compared with those obtained at 8 weeks *p.i*. MGDG is metabolized by *Streptococcus pneumoniae*, with conversion between DGDG and MGDG (57). It could be considered that certain enzymatic reaction processes in *S. pneumoniae* might also be present in other bacterial genera; thus, lipid characterization in representative gut microbiota might contribute to the deciphering of the bacteria associated with the glycerolipid alteration observed here. Moreover, PE, which was decreased at 16/24 weeks *p.i*., was distributed in the representative human gut microbe *Alistipes finegoldii* in the phylum *Bacteroidetes* (58). This is not surprising because of the age-dependent decrease in F/B ratio observed.

Among other lipids, coenzymes (i.e., coenzyme Q9H2) showed decreased levels at 16 weeks *p.i*. compared with 8 weeks *p.i*. Considering the age-dependent reduction in plasma glucose detected in broiler chickens (59), the decreased levels of coenzymes might be part of the age-dependent dynamics.

In summary, we demonstrated that the long-term breeding of laying hens decreased *C. jejuni* colonization in the cecum after experimental infection. Comparative analyses of the alterations of gut microbiota and lipid components at 16 weeks *p.i*. or later unveiled possible negative associations between *C. jejuni* and several gut microbiota, such as those in the genera *Blautia* and *Clostridium* at younger or older age, respectively. It is likely that the chicken generally reaches maturity and starts laying eggs from 21 weeks old on average (60), which is close to the age at 16 weeks *p.i*. (18 weeks of age) when we observed the alterations in *C. jejuni* colonization, microbiota, and lipid compositions in the gut of laying hens. Thus, it could be considered that the altered phenomenon's observed in this study might be mainly due to certain host physiological change(s) accompanied with the host maturation. Our future study of the interplay between these gut microbiota and bile acid metabolism, as well as *C. jejuni* colonization, in laying hens is expected to improve our understanding of the possible interactions between these parameters, thereby leading to the discovery and establishment of control strategies for the reduction of *C. jejuni* intestinal carriage at poultry-production stages.

## Data Availability Statement

The datasets presented in this study can be found in online repositories. The names of the repository/repositories and accession number(s) can be found below: https://www.ncbi.nlm.nih.gov/genbank/, DRA009061.

## Ethics Statement

The animal study was reviewed and approved by Animal Welfare and Ethical Committee of the National Institute of Health Sciences.

## Author Contributions

HA conceived and designed the study. HA, TN, SY, KI, and JK performed and analyzed the experiments, and HA wrote the manuscript. IK, JK, YT, and SM read and edited the manuscript. All authors contributed to the article and approved the submitted version.

## Conflict of Interest

The authors declare that the research was conducted in the absence of any commercial or financial relationships that could be construed as a potential conflict of interest.
