# Supplementary material for: Long-Term Grow-Out Affects Campylobacter jejuni Colonization Fitness in Coincidence With Altered Microbiota and Lipid Composition in the Cecum of Laying Hens
Source: Front Vet Sci. 2021 Jun 18;8:675570. doi: 10.3389/fvets.2021.675570 (PMC8249580; doi:10.3389/fvets.2021.675570)
Supplement: Supplementary file 1 [file Data_Sheet_1.pdf]

Supplementary Material

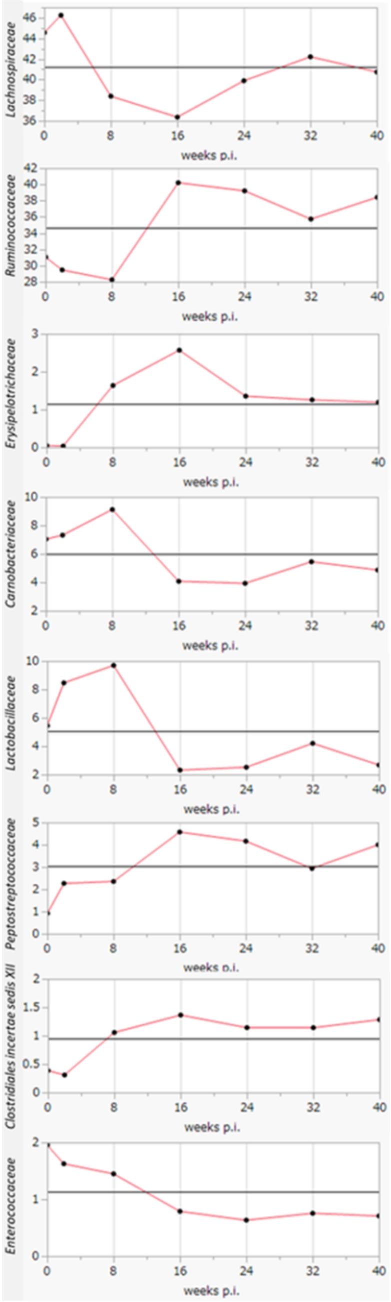

Supplementary Figure 1. Time-dependent dynamics of representative bacterial families detected in ceacal samples of laying hens. The Y-axis represents the relative abundance (as a percentage).

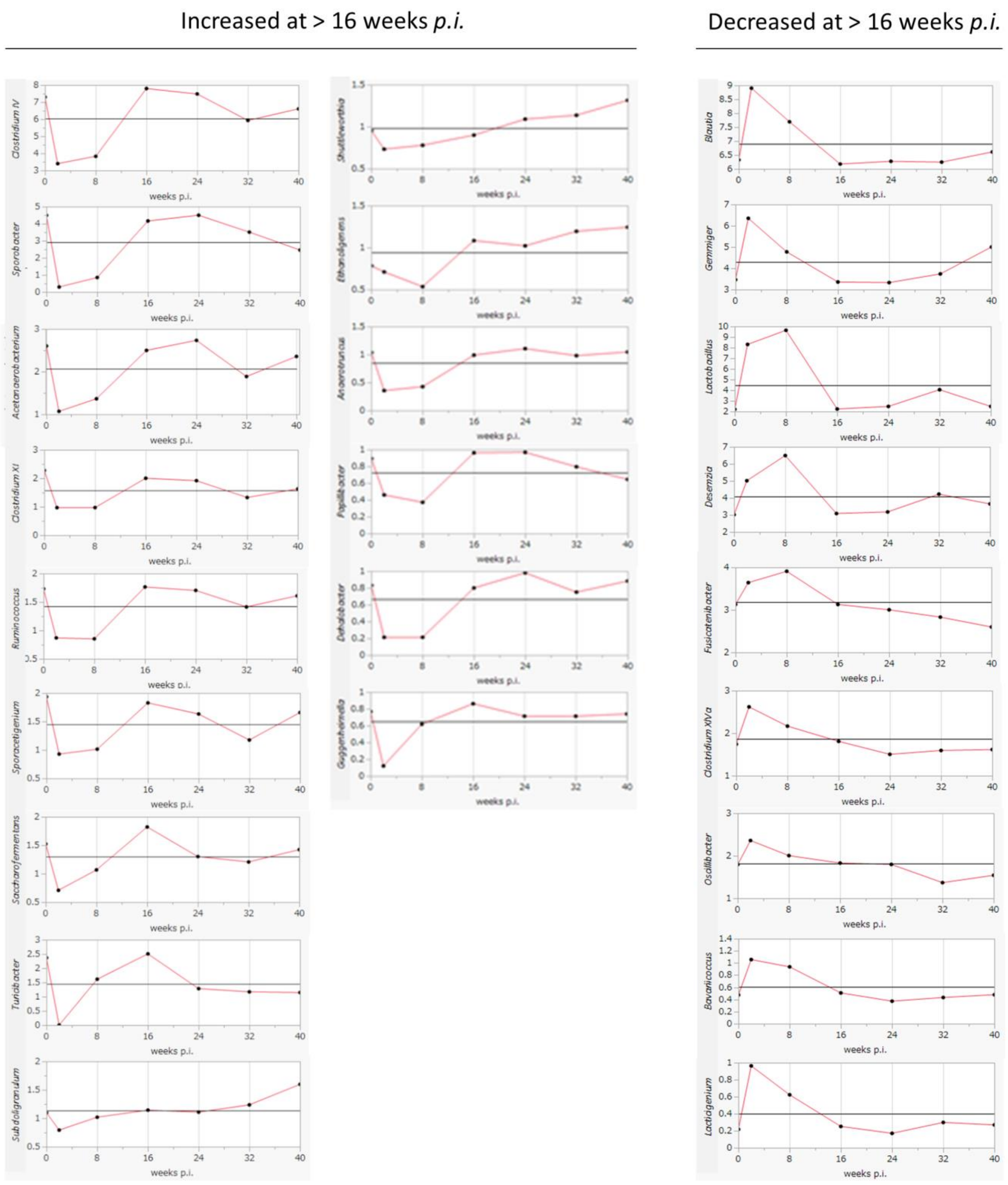

Supplementary Figure 2. Time-dependent dynamics of representative bacterial genera exhibiting a relative abundance >0.50% on average, as detected in ceacal samples of laying hens. Each genera are indicated in Table 1. The Y-axis represents the relative abundance (as a percentage).

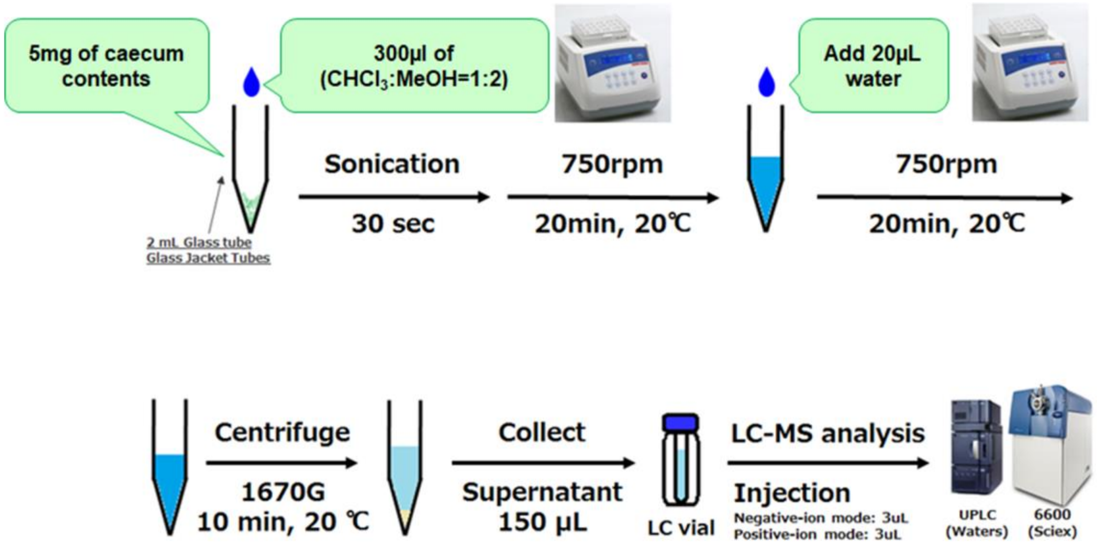

Supplementary Figure 3. Workflow of sample preparation for lipidome analysis.
